# Synthesis and Characterization of Polyvinyl Chloride Matrix Composites with Modified Scrap Iron for Advanced Electronic, Photonic, and Optical Systems

**DOI:** 10.3390/nano12183147

**Published:** 2022-09-11

**Authors:** Syed Usama Mauood Hashmi, Muhammad Aamir Iqbal, Maria Malik, Muhammad Tariq Qamar, Maham Khan, Abu Zahid, Md. Rasidul Islam, Mohammed Al-Bahrani, Kareem Morsy, Wen-Cheng Lai

**Affiliations:** 1 Department of Chemistry, Forman Christian College, Lahore 54600, Pakistan; 2 School of Materials Science and Engineering, Zhejiang University, Hangzhou 310027, China; 3 Centre of Excellence in Solid State Physics, University of the Punjab, Lahore 54590, Pakistan; 4 Department of Computer Science, Lamar University, Beaumont, TX 77705, USA; 5 Department of Electrical and Electronic Engineering, Bangamata Sheikh Fojilatunnesa Mujib Science and Technology University, Melandah, Jamalpur 2012, Bangladesh; 6 Air conditioning and Refrigeration Techniques Engineering Department, Al-Mustaqbal University College, Babylon 51001, Iraq; 7 Biology Department, College of Science, King Khalid University, Abha 61421, Saudi Arabia; 8 Bachelor Program in Industrial Projects, National Yunlin University of Science and Technology, Douliu 640301, Taiwan; 9 Department of Electronic Engineering, National Yunlin University of Science and Technology, Douliu 640301, Taiwan

**Keywords:** scrap iron, modified Fe_2_O_3_, co-precipitation, PVC films, optical and thermal properties

## Abstract

In this study, Fe_2_O_3_ powder was synthesized using the co-precipitation method from scrap iron, which was then treated with varying concentrations of copper. Afterwards, the modified Fe_2_O_3_ was reinforced in the PVC matrix by using the solution-casting method to synthesize PVC composite films, which were subjected to a UV-visible spectrophotometer, a Fourier transform infrared spectrophotometer, an X-ray diffractometer, and a thermal gravimetric analyzer to evaluate the optical, chemical, structural, and thermal properties. FTIR analysis reveals the formation of the composite through vibrational bands pertaining to both components present, whereas no significant changes in the XRD patterns of PVC were observed after the doping of modified iron oxide, which reveals the compatibility of fillers with the PVC matrix. The optical properties of the copper-doped iron oxide-PVC composites, including absorbance, refractive index, urbach energy, and optical as well as electrical conductivity are measured, and show an increase in optical activity when compared to the pure PVC compound. Moreover, the increased thermal stability of the synthesized composite was also observed and compared with conventional compounds, which, in accordance with all the other mentioned properties, makes the copper-dopped iron oxide-PVC composite an effective material for electronic, photonic, and optical device applications.

## 1. Introduction

Polymer composites are the most dynamically developed materials, owing to their ability to improve mechanical, thermal, and barrier properties of the composite, including their cost-effective nature [1]. Polyvinyl chloride (PVC) is one of the common polymer compounds having high thermal stability and processibility compared to other polymer compounds such as polyethylene (PE), polyamide (PA), and polypropylene (PP). These properties of PVC can be reinforced by the addition of powder particles known as nanofillers, in small quantities to enhance the material properties such as optical clarity and self-passivation, along with an increased resistance to oxidation and ablation. These polymer composites have wide applications in coatings, automotive parts, and flame retardance [2]. Powder fillers have a substantial variety in their shapes, sizes, chemical structures, as well as forms, and possess essential properties of various compounds that can be used as potential fillers. They are inflexible materials, immiscible with the matrix in both liquid and solid states, classified as inorganic or organic substances based on different varying chemical functions. Magnetite, maghemite, and hematite are all forms of iron that exist in nature, and researchers have explored that with the decrease in the size of maghemite, the vacancy order also decreases, so that at 20 nm, there is no vacancy order present. However, above the Curie temperature of 956 k, hematite shows paramagnetic behavior, along with the phase transition properties at 260 k to an antiferromagnetic state and is weakly ferromagnetic. The parameters include the extent of cation substitution, particle size, and crystallinity to define the magnetic behavior of hematite. Moreover, while decreasing the particle size of hematite, the Morin temperature also decreases and becomes suppressed at a particle size range of 8–20 nm. Therefore, for these reasons, hematite acts as the best filler material [3].

PVC was chosen for polymeric matrix nanocomposites (PMNC) from the classification of composites such as metal, ceramic, and polymeric matrix nanocomposites (PMNC) because PVC is the major thermoplastic that exhibits remarkable mechanical, optical, thermal, and flammable properties when incorporated with nanofillers via solution casting, in situ polymerization, or mixing techniques [4]. The chemical and biological resistance of PVC is considerable, and it has enhanced flexibility and durability [5,6]. In this study, copper-doped iron oxide has been used as a filler in PVC film to enhance the strength, specific compresses, specific flexural modulus, and density of the PVC membrane, and it explores the synthesis of copper-doped iron oxide nanocomposite into the repeating unit of PVC polymer as a matrix to further evaluate the electrical and optical properties of the synthesized material and their possible applications in commercially available devices of advanced technology. In recent years, the electrical and optical properties of polymers have attracted much attention in optical device technology due to their unique polarization, interference, reflection, and antireflection properties [7,8,9]. For the following reasons, optical fibers and reflected coating applications require advanced optical materials to cover a wide range of wavelengths; as the optical properties of materials are associated with their atomic structure and electronic structure, which can be modified by suitable material doping, the desired optical properties can be achieved [10]. In the present study, scrap iron as a precursor is used to synthesize powder iron oxide nanoparticles, which are doped with a minor amount of copper to further synthesize copped-dopped iron oxide-PVC composite film, which is an economical process to encounter the efficiency-based problems of electronic and photonic devices owing to the lower bandgap value of the reference material PVC. The atomic absorption spectrophotometer was also reported to be used in this process to detect the exact amount of iron to ensure its excellent accuracy [11].

The use of scrap iron in the field of technology is very fruitful in generating very effective powder particles and will solve the economic problems in the near future. The modified materials have smaller bandgaps than their reference materials such as PVC and produce fruitful results in the field of electronic devices that emit light. In this way, the problem of the less efficient operation of these electronic devices can be solved. This study reports the synthesis and characterization of a copper-doped iron oxide-PVC (CDF) composite film using scrap iron solution as a precursor by adopting a co-precipitation method to explore the potential of these films for advanced electronic, photonic, and optical system applications.

## 2. Materials and Methods

### 2.1. Materials

In this study, hydrochloric acid (HCl), potassium hydroxide (KOH), scrap iron, copper chloride (CuCl_2_·5H_2_O), Triton-X 100, and ethanol were used as chemical regents. All of the chemicals were purchased from Sigma Aldrich, distributors from Lahore, Pakistan, except for HCl, and were of the highest purity.

### 2.2. Equipment

A water bath sonicator, hot plate, muffle furnace, weight balance, and atomic absorption spectrometer were the apparatus components used, and were available at the Department of Chemistry, Forman Christian College University, Lahore, Pakistan.

### 2.3. Methodology

**Figure 1:**
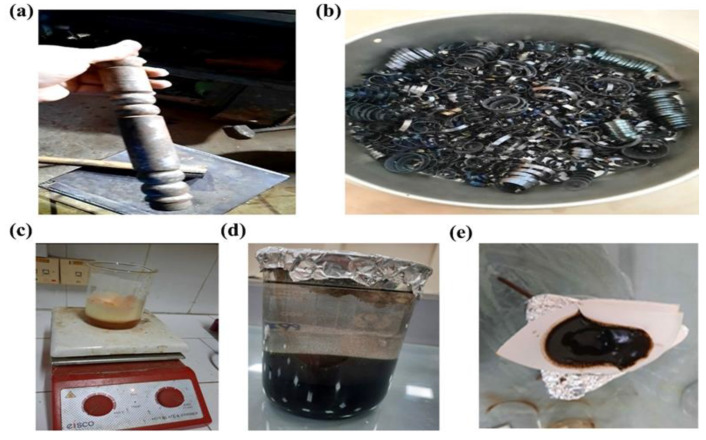
Synthesis illustration, (**a**) iron rod, (**b**) scrap iron, (**c**) solution of copper chloride and iron chloride, (**d**) precipitates of CDF, and (**e**) precipitates of iron oxide.

The synthesis of copper-doped iron oxide is accomplished by using scrap iron and copper chloride as precursors via the co-precipitation method, which is further reinforced with polyvinyl chloride by using a solution-casting method to get PVC-based composites. The detail of the synthesis methodology, which involves scrap iron digestion, synthesis of pure and modified iron oxide, and preparation of PVC-films, is discussed below and displayed in Figure 1.

#### 2.3.1. Scrap Iron Digestion

For scrap iron synthesis, the iron rod was used to crush the scrap iron (Figure 1a,b) into tiny particles of iron and dissolved into 800 mL of HCl for 24 h, which was then filtrated before being subjected to an atomic absorption spectrophotometer (AAS) used to analyze the exact amount of iron present in the filtered solution. Furthermore, the filtered solution was diluted 100 times and then again subjected to AAS for efficient results. Moreover, three other samples of copper-doped iron oxide were prepared via the co-precipitation method by varying copper concentrations while maintaining the equal molarity of both copper and iron oxide solutions, which were further analyzed through AAS. The samples with 1%, 3%, and 5% copper concentrations in copper-doped iron oxide (Fe_2_O_3_) samples were prepared, while one sample of pure iron oxide was also used as a reference sample. The concentration of iron present in 1 mL of iron chloride was analyzed by AAS at 228.734 ppm, whereas diluting it 100 times resulted in 22873.4 mg/L or 22.8734 g/L, having a molar concentration of 0.4096 M. Furthermore, for Cu doping to Fe2O3, a copper chloride precursor of the same molarity, 0.4096 M, was used.

#### 2.3.2. Synthesis of Pure and Modified Iron Oxide

For the synthesis of iron oxide, first, a 100 mL solution of iron chloride was added to the copper chloride solution by constant stirring and heating on a hot plate at a temperature of 80–85 °C, which was followed by 4 mL of Triton X 100 as a surfactant to the solution. Afterward, a 5-molar solution of KOH was added dropwise through the burette. This process is known as hydrolysis, and the solution became hydrolyzed after consuming 170 mL of the base, appearing in the form of precipitates, which were then washed with distilled water to achieve a neutral pH. Precipitates of Cu-doped iron oxide (CDF) and pure iron oxide are displayed in Figure 1c–e, respectively.

The wet precipitates were dried in the oven at 100 °C for 5 h before being ground to be used further for calcination. The sample was ground again with a mortar and pestle after 4 h of calcination at 400 °C for the formation of pure iron oxide. The other three samples of varying Cu concentrations of 1%, 2%, and 3% were also prepared by adopting a similar procedure.

#### 2.3.3. Synthesis of PVC composites films

**Figure 2:**
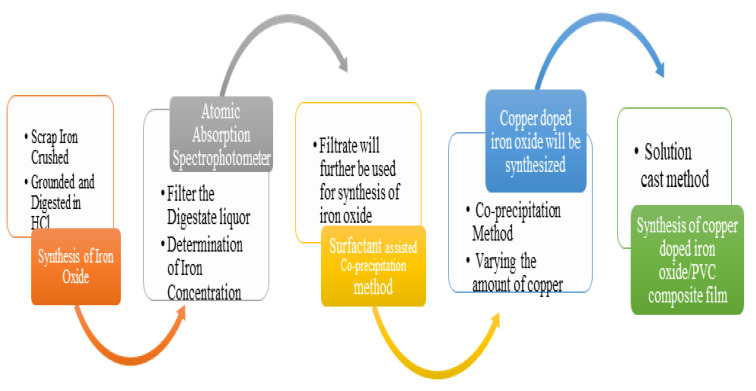
Flow sheet diagram of synthesis of Cu-doped iron oxide/PVC-composite films.

After synthesizing doped materials, four samples of 1%, 3%, and 5% concentrations of copper-doped iron oxide-PVC (CDF) composites along with a pure PVC-iron oxide sample were prepared by using the solution-casting method. For the synthesis of 1% CDF-PVD composite film, 1 g of PVC was dissolved into 50 mL of tetrahydrofuran (THF) in a beaker at constant heating and stirring for 7 h at 80–85 °C. After that, PVC containing THF was poured into a petri dish, where PVC film was synthesized after the evaporation of THF in 10 to 12 h, which was then separated from the petri dish with the help of a cutter. Similarly, the other composite films with 3% and 5% Cu-concentrations were also prepared by following the same procedure [12,13]. The flow sheet diagram of the overall process is presented in Figure 2.

## 3. Results and Discussion

This section explains the optical results obtained from UV visible spectroscopy, including direct and indirect bandgap, transmittance, optical and electrical conductivity, absorption, and refractive index, as well as the structural properties of the sample using X-ray diffraction pattern, chemical composition using FTIR, and thermal analysis of prepared PVC-based composites. Furthermore, this section reveals novel outcomes and explores the potential of these PVC films for different device-based applied applications.

### 3.1. X-ray Diffraction Analysis

**Figure 3:**
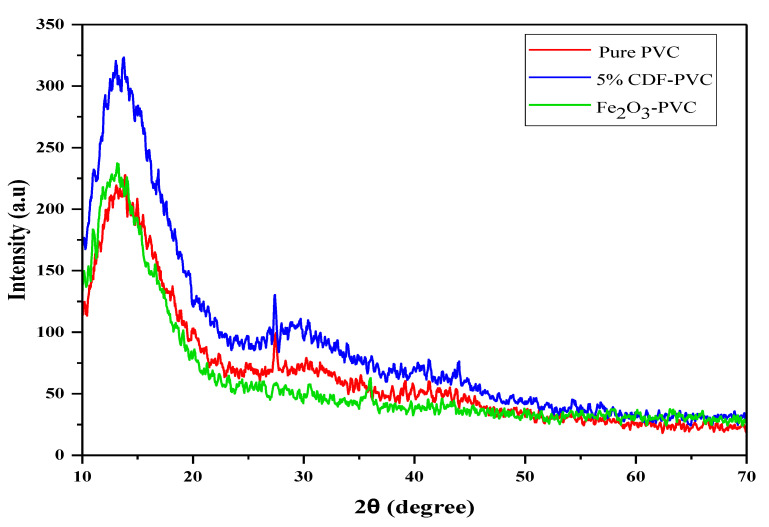
The comparison of XRD patterns of PVC and its composites.

An X-ray diffraction analysis can be used to describe the material’s structural parameters. The sharp peaks of XRD define the crystallinity, while the broad peaks show the amorphous nature of a sample. In the present study, different samples of Cu-doped Fe_2_O_3_ with varying concentrations of Cu were prepared, but we investigated only three main samples for XRD analysis. For this purpose, an X-ray diffractometer was used with Cu k radiation at a specific wavelength of λ = 1.54 Å. The obtained XRD patterns for PVC, Fe_2_O_3_ -PVC, and 5% CDF-PVC are depicted in Figure 3. The XRD pattern of pure PVC confirms its amorphous nature with no prominent sharp peak, but a broad peak in the region of 2θ in the range of 10° to 25° appears as previously reported in the literature. Likewise, after its modification with 5% Cu concentration, four minor peaks were observed in the pattern at 2θ values of 13.89°, 27.34°, 41.3°, and 44.21°, respectively, confirming the semi-crystalline nature of the modified PVC composite [14]. The Fe_2_O_3_-PVC has four minor peaks in the diffraction pattern at 2θ values of 13.29°, 17.16°, 36.34°, and 53.7°, respectively, showing that this material has the same semi-crystalline nature as its counter parts. The band present under the range of 10.24° to 16.66° in PVC, 5% CDF-PVC, and Fe_2_O_3_-PVC is almost the same in these three samples, indicating the strong interaction between CDF and PVC. This is the best evidence of the aggregate formation in 5% CDF-PVC and Fe_2_O_3_ -PVC [15].

### 3.2. Fourier Transform Infrared (FTIR) Spectroscopy

**Figure 4:**
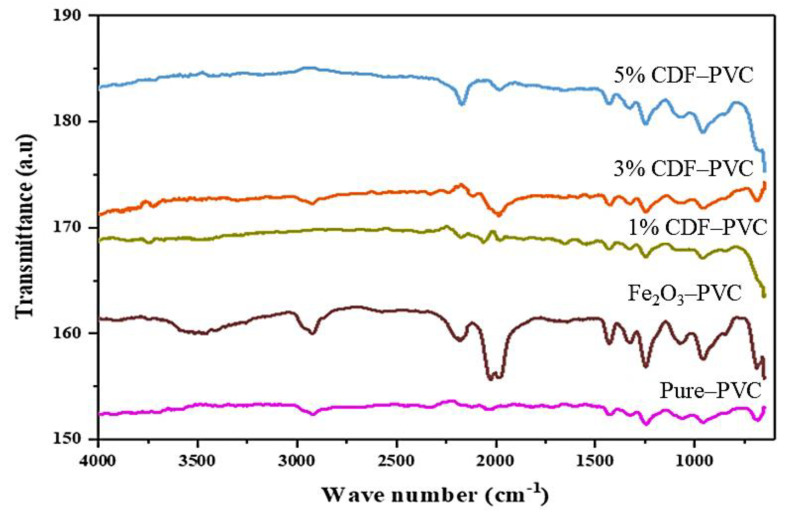
A comparison of the FTIR spectra of PVC and its composites.

FTIR spectroscopy analysis can be used for the detection of various functional groups present in nanomaterials, as well as to identify the formation style of composites. The combined spectra of synthesized PVC composites give different vibrational peaks at different wave numbers depending on the functional groups present, as shown in Figure 4. The spectrum of PVC shows numerous vibrational peaks at different wave numbers, including a vibrational band at 684 cm^−1^ that indicates the presence of the C-Cl functional group, while a band at 961 cm^−1^ depicts the CH_2_ group, 1084 cm^−1^ shows the presence of the C-C bond group, 1438 cm^−1^ represents the wagging of the CH_2_ group, and the 2930 cm^−1^ wavenumber shows the presence of the C-H group of CHCl. On the other hand, the composites of PVC with different Cu-concentration doped iron oxide reveal bands at 654 cm^−1^, 961 cm^−1^, 1254 cm^−1^, 1438 cm^−1^, 1931 cm^−1^, 2176 cm^−1^, 2038 cm^−1^, and 2176 cm^−1^, confirming the formation of PVC composites. The band revealed at 654 cm^−1^ indicates the presence of halo compounds, while at 961 cm^−1^ transmittance peaks, the alkene bond was present with strong C=C bending. However, an alkyl aryl ether group with strong C-O stretching was present at the 1254 cm^−1^ transmittance band along with a carboxylic acid group having O-H bonding at the 1438 cm^−1^ band. Alongside, aromatic compounds with C-H banding, alkyne groups, and isothiocyanate groups were present at 1931 cm^−1^, 2176 cm^−1^, and 2038 cm^−1^ transmittance peaks, respectively. The formation of PVC composites confirms all these functional groups’ presence. Furthermore, the peaks at 1069 cm^−1^ and 1254 cm^−1^ represent the presence of C-H in the CH-Cl stretching of PVC, while at 1438 cm^−1^ and 2939 cm^−1^, they represent the wagging behavior of the CH_2_ group in composites [14]. Table 1 summarizes all of the data for all of the exact peaks of the prepared composites, along with their numbers and peak assignments.

In the case of the spectrum of Fe_2_O_3_-PVC composite, the bands at 700 cm^−1^ and 684 cm^−1^ indicate the asymmetric stretching of the Fe–O bond. The peaks at 1438 cm^−1^, 1654 cm^−1^, and 1454 cm^−1^, present in Fe_2_O_3_-PVC, 1% CDF-PVC, 3% CDF-PVC, and 5% CDF-PVC, indicate the complexity of the polymer filler showing the Cu-OH bonding. In 3% CDF-PVC, 5% CDF-PVC, and Fe_2_O_3_-PVC, a band at 2930 cm^−1^ indicates the vibrational peak of PVC C-H of CHCl in composites [14,16,17,18].

### 3.3. UV-Visible Spectroscopy

**Figure 5:**
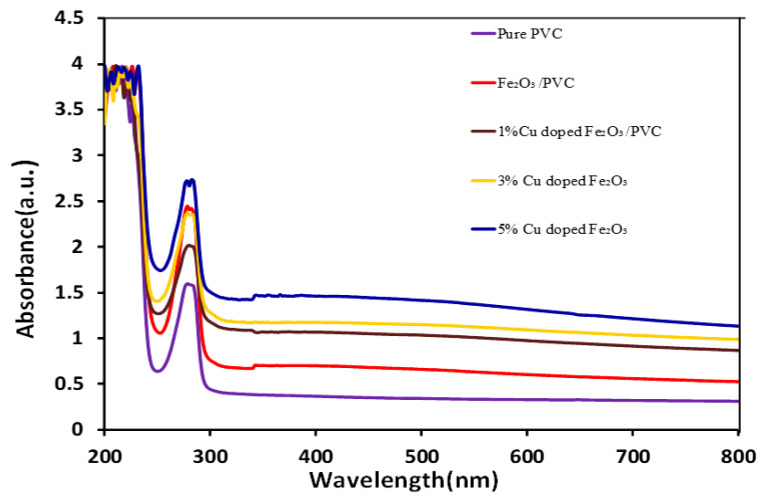
A comparison of absorption spectra of PVC and its composites.

A UV-visible spectrophotometer analysis can be used to explore all optical properties of the prepared samples, including direct and indirect bandgap, transmittance, optical conductivity, electrical conductivity, and refractive index. Taha et al. [19] reported the same procedure to investigate the optical properties of PVC-Al_2_O_3_ films. They observed that PVC doped with Al_2_O_3_ with different percentages in increasing order absorbed more light than intrinsic pristine PVC [19]. Herein, we observed the optical spectra for pure PVC, pure Fe_2_O_3_-PVC composite, and Cu-dopped Fe_2_O_3_-PVC composite with varying concentrations of Cu of 1%, 3%, and 5%. The spectra shows that pure PVC absorbed a lower amount of energy in the UV region than PVC doped with varying concentrations of Cu, the composites absorbed more energy in the UV region. Here, 5% Cu-doped Fe_2_O_3_-PVC absorbed more light in the UV region than its counterparts, as depicted in Figure 5. The increasing order of absorbance is observed as: pure PVC < 1% CDF-PVC < 3% CDF-PVC < Fe_2_O_3_-PVC < 5% CDF-PVC.

The patterns in Figure 5 indicate an increase in absorption with increasing the concentration of Cu-doping in the investigated composite. The increase in absorption below 255 nm depicts the C-Cl bond. However, from 266 to 280 nm, there was maximum absorbance, which started to decrease after 280 nm. Pristine PVC has less absorption in both the UV and visible regions, but after doping with a 5% concentration of Cu, the composite presented high absorbance in both the visible and UV regions. However, a 3% Cu-concentrated CDF-PVC composite absorbed more light in the visible region than the UV region, while a 1% Cu-concentrated CDF-PVC composite showed excellent activity in the visible region but less in the UV region. The absorbance of Cu-doped Fe_2_O_3_ increases with increasing the Cu-doping content, and a maximum absorbance was observed for 5% Cu-doped Fe_2_O_3_. Additionally, Fe_2_O_3_/PVC films show a higher absorbance than the 1% and 3% Cu-doped Fe_2_O_3_, but less than the 5% Cu-doped Fe_2_O_3_. These results are in accordance with the extinction coefficient values for which the maximum value corresponds to 5% Cu-doped Fe_2_O_3_. From the above discussions and graphical analysis, it is clear that individual PVC did not show activity in both visible and UV regions, so external modifications are necessary for its enhanced activity. However, more absorption of prepared PVC composite films in UV and visible regions than pristine PVC indicates that iron and Cu-doped iron oxide attached gently to the surface of PVC. Table 2 summarizes all the direct and indirect bandgap energy values.

### 3.4. Direct Bandgap of PVC Composites

**Figure 6:**
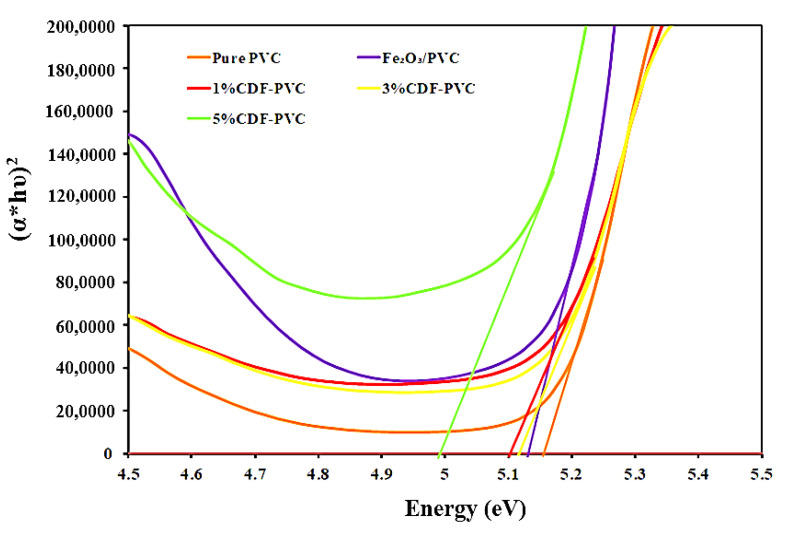
A comparison of the direct bandgap of PVC films and their composites.

The spectra of the direct bandgap of pure PVC, Fe_2_O_3_-PVC, 1% CDF-PVC, 3% CDF-PVC, and 5% CDF-PVC composite is presented in Figure 6, which confirms the bandgap energy of pure PVC as 5.15 eV, in accordance with reported literature, wherein pure PVC has a direct bandgap of 5.15 eV [20]. However, the modified Fe_2_O_3_-PVC composite with Cu concentrations of 1%, 3%, and 5% depicts a reduction in the bandgap energy compared to pristine PVC, indicating the aggregates between the powder materials and PVC, which means that conduction and valance band are close to each other in the modified Fe_2_O_3_-PVC composite, making them act as an insulator. The concentrated Cu-doped composite, 5% CDF-PVC, has a bandgap of 4.85 eV, while Fe_2_O_3_-PVC has a greater bandgap energy than 5% CDF-PVC, 5.13 eV, confirming that PVC reinforced with pure iron shows the best activity for photonic, electronic, and optical applications.

### 3.5. Indirect Bandgap of PVC Composites

**Figure 7:**
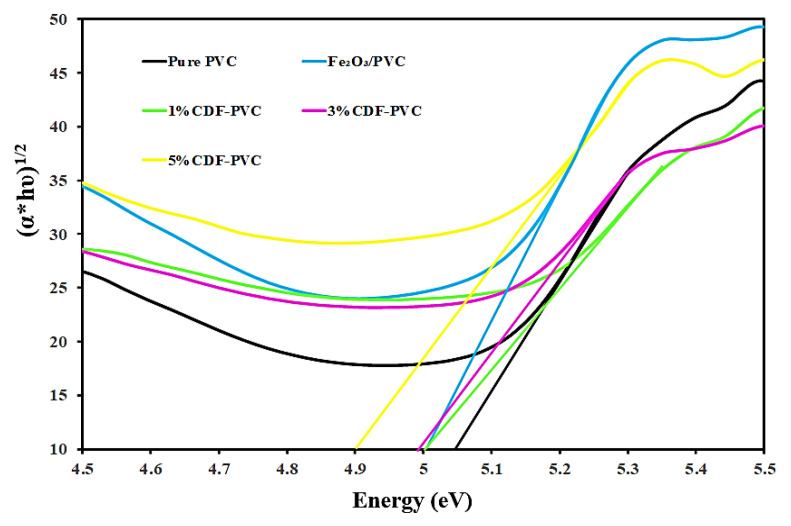
A comparison of the indirect bandgap of PVC films and their composites.

The graph between square-root of absorbance and incident photon energy [(α*hυ)^1/2^ vs. hυ] was used to explore the indirect bandgap nature of the fabricated films. It was observed that the pure PVC has an indirect bandgap energy of 5.05 eV, while both the Fe_2_O_3_-PVC and 3% CDF-PVC composites showed 4.99 eV as an indirect bandgap, as shown in Figure 7. Furthermore, the synthesized PVC composites have an indirect bandgap energy less than their direct bandgap energy, so they have a huge potential to be employed in photocatalysis and nanophotonics [16,19,21].

### 3.6. Transmittance

**Figure 8:**
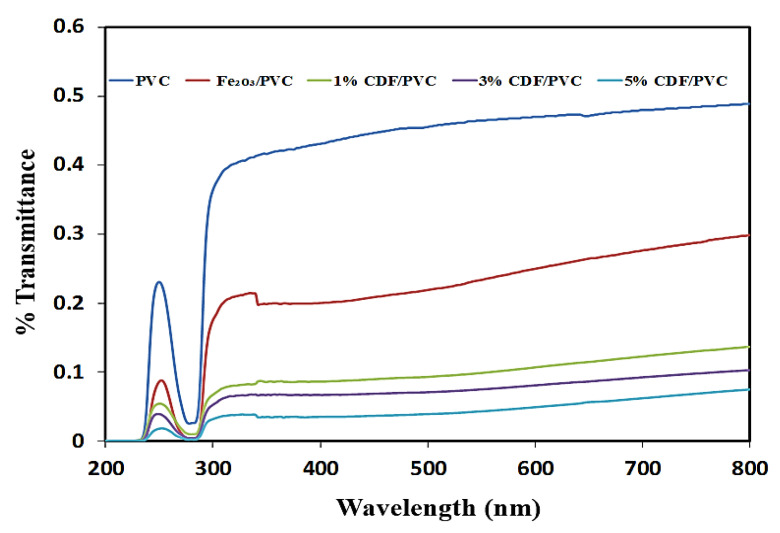
A comparison of transmittance spectra of PVC films and their composites.

Transmittance is an optical property being investigated which shows the part of incident light that is transmitted from the material. The present study explains the transparency of pure PVC, Fe_2_O_3_-PVC, 1% CDF-PVC, 3% CDF-PVC, and 5% CDF-PVC, as demonstrated in the spectrum of Figure 8. The spectra indicate that pure PVC has more transparency than its composites, while Fe_2_O_3_-PVC has less transparency than pure PVC. However, 1% CDF-PVC has less transparency than both Fe_2_O_3_-PVC and pure PVC. Moreover, the 3% CDF-PVC is less transparent than both the pure PVC and the 1% CDF-PVC and Fe_2_O_3_-PVC, while the 5% CDF-PVC has the minimum transparency of its counterparts. This reduction in transmittance indicates that scattering of light occurs from the samples, confirming the excellent aggregate formation of Cu-doped iron oxide with PVC. The literature also confirmed the maximum transparency of pure PVC. Furthermore, these spectra clearly show that 5% CDF-PVC has more aggregation of CDF with PVC than other composites, wherein all the synthesized composites absorbed the maximum UV radiation in the range of 252–254 nm. In addition, percentage transmittance can be used to evaluate the exact amount of scattered light of a certain wavelength, confirming that no light scattering occurred for pure PVC; however, the scattering effect was maximum for the 5% CDF-PVC composite.

### 3.7. Extinction Coefficient

**Figure 9:**
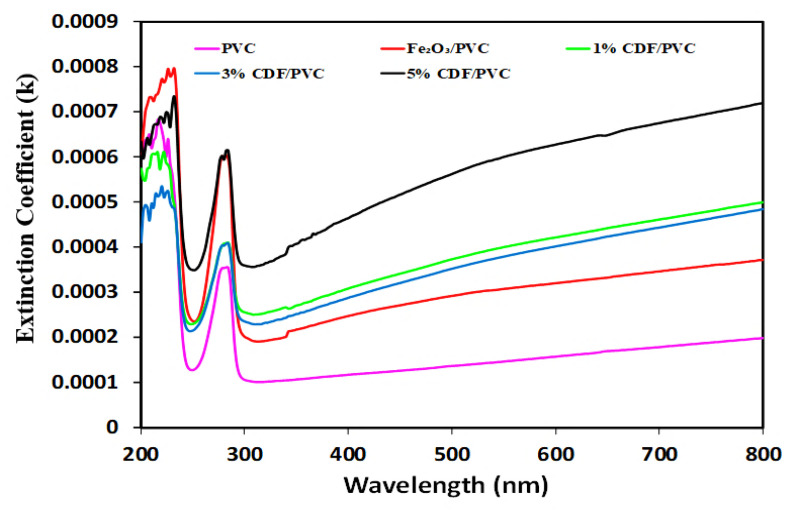
A comparison of extinction coefficient spectra of PVC and its composites.

The extinction coefficient is the property that exactly predicts the behavior of the material when studying its absorbance. For the investigated samples, it was calculated by adopting the formula, *K* = αλ/4π, wherein, α is the ratio of the thickness of film to absorptance. The extinction coefficient spectra of pure PVC, Fe_2_O_3_-PVC, 1% CDF-PVC, 3% CDF-PVC, and 5% CDF-PVC are demonstrated in Figure 9, which shows that the 5% CDF-PVC composite has a higher extinction coefficient than the 1% CDF-PVC composite, while pure PVC has a lower value of extinction coefficient than its counterparts. Therefore, pure PVC absorbed less energy in the visible region, while 5% CDF-PVC absorbed more energy in the visible region than the 3%, 1%, and Fe_2_O_3_-PVC.

### 3.8. Refractive Index

**Figure 10:**
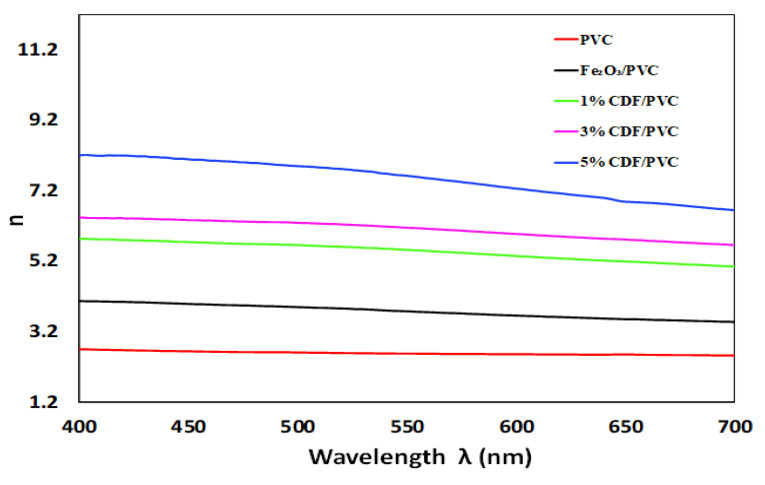
A comparison of the refractive index of PVC and its composites.

The refractive index determines the reduction in the speed of incident light and its dependence on a medium, denoted by *n*(*ω*) The present study explores the refractive index of pure PVC, Fe_2_O_3_-PVC, 1% CDF-PVC, 3% CDF-PVC, and 5% CDF-PVC, as shown in Figure 10. It is clear that the refractive index has increased with increasing the Cu doping concentration in Fe_2_O_3_-PVC composite, owing to the condensation of ceramic molecules into larger clusters. The increase in the refractive index of materials finds its potential applications in optical devices and photovoltaic devices, such as photonic crystals, Bragg gratings, and solar cells [22]. This study clearly shows that 5% CDF-PVC material is efficient for the abovementioned applications due to its high refractive index. The order of refraction is observed as: 5% CDF-PVC > 3% CDF-PVC > 1% CDF-PVC > Fe_2_O_3_-PVC > pure PVC.

### 3.9. Urbach Energy

The Urbach energy, also known as the Urbach edge, is a quantity that is frequently designated by the letters E_0_ and is used to measure energetic disorder in semiconductor band edges. It is determined by fitting the absorption coefficient to an exponential curve as a function of energy and is frequently used to describe electron transport in semiconductors with structural disorder, such as hydrogenated amorphous silicon [23]. It is reported that the localized states produced within the bandgap are due to an increase in Urbach energy with an increase in doping concentration. The inverse slope of the straight-line equation describes the Urbach energy in numeric form. The spectra of synthesized samples show that PVC has a lower amount of Urbach energy than 5% CDF-PVC, which has the maximum value of it, compared to the other composites. Table 3 summarizes all the Urbach energy values.

### 3.10. Optical Conductivity

**Figure 11:**
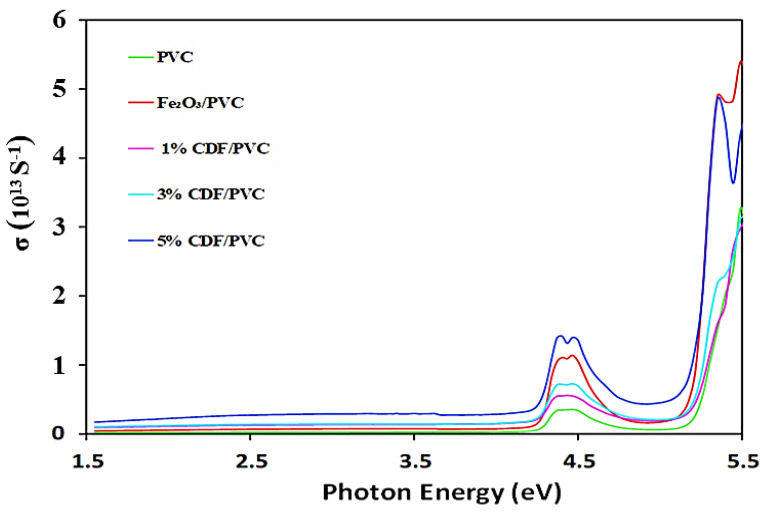
A comparison of optical conductivity spectra of PVC and its composites.

The term optical conductance, which can be derived from the imaginary component of the dielectric function and is sensitive to charged responses, refers to the electrical transmission expansion to high optical frequencies. In this study, PVC spectra showed very low optical conductivity, while Cu-doped PVC composites showed a higher level of optical conductivity than pure PVC. The spectrum presented in Figure 11 clearly shows the high optical conductivity of 5% CDF-PVC, relating to a smaller direct and indirect bandgap. A direct relation between optical conductance and incident photon energy is observed, such that the conductivity increases with an increase in incident radiation. The maximum optical conductance corresponds to Fe_2_O_3_-PVC at a high incident photon energy, while the lowest conductance can be seen for pure PVC. It is also observed that higher incident radiation yields high optical conductance, as the direct band transitions increase with an increase in incident energy.

### 3.11. Electrical Conductivity

**Figure 12:**
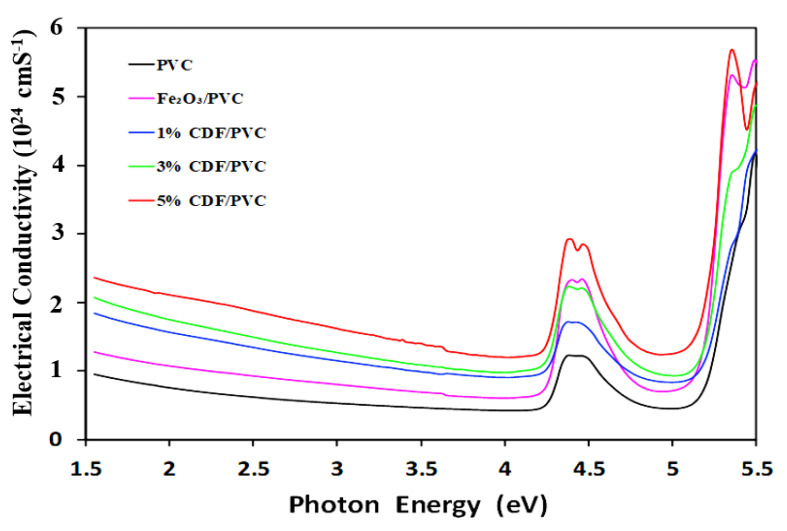
A comparison of electrical conductivity spectra of PVC and its composites.

A material’s electrical conductivity can be determined by observing how easily an electric current flow through it. Electrical resistivity, on the other hand, gauges how much a substance resists the flow of electricity. The electrical conductivity of PVC and PVC composites is presented in Figure 12, illustrating that pure PVC has a lower amount of electrical conductivity, hence proving it a good insulator. In order to increase the electrical conductivity, PVC was modified with copper-doped iron oxide with different concentrations, further revealing the fact that a highly concentrated PVC composite is the best conductor of electricity compared to other synthesized PVC composites. The order of electrical conductivity is observed as: 5% CDF-PVC > Fe_2_O_3_-PVC > 3% CDF-PVC > 1% CDF-PVC > pure PVC, while higher incident photon energy yields higher electrical conductance, depicting the conducting nature of Co-doped PVC and Fe_2_O_3_-PVC.

### 3.12. Thermal Gravimetric Analysis

**Figure 13:**
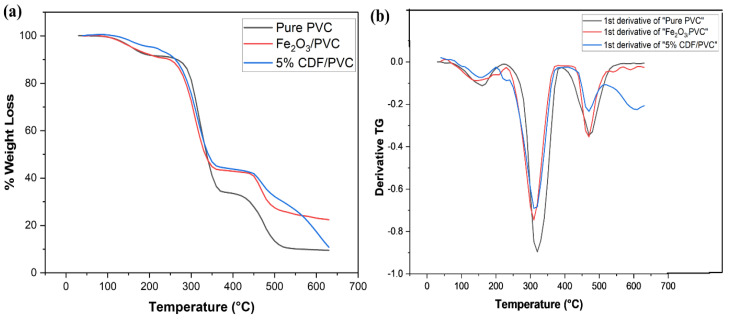
(**a**) A comparison of thermogram of PVC, Fe_2_O_3_, and 5% CDF–PVC, and (**b**) comparison of first derivative of PVC, Fe_2_O_3_–PVC, and 5% CDF–PVC.

Thermal gravimetric analysis (TGA) indicates the thermal stability and degradation within a wide range of temperatures for various potential device applications in accordance with the material’s thermal stabilities. The results of TGA analysis for the synthesized PVC composite are presented in Figure 13 in the form of a spectrum. These composites convert their physical state from glass to semi-liquid at varying temperatures. The spectrum of pure PVC shows the two steps of degradation. In the first step, hydro choleric acid is released at the temperature range of 270 °C to 370 °C with a major weight loss of 57%, while the second phase of PVC degradation starts at the temperature range of 440 °C. In the second phase, different cycles of conjugate polyethylene occur to obtain different aromatic compounds. In other words, we can say that cross-linking, aromatization, and isomerization occur. However, a major weight loss of synthesized Fe_2_O_3_-PVC and 5% CDF-PVC was analyzed under the range of 240–360 °C and 250–360 °C with a weight loss of 48.72% and 46.82%, respectively (Figure 13a). This decrease in weight loss of both materials indicates a breakdown of organic molecules from the surface of powder particles. Here, T_10_ of PVC, Fe_2_O_3_-PVC, and 5% CDF-PVC were calculated as 267 °C, 248 °C, and 263 °C, respectively, presenting the thermal stability of samples with the decreased value of temperature for modified PVC composite because of the filler material that pulled Cl from PVC. However, the Cu-doped PVC composite showed a sudden increase in T_10_, indicating that 5% CDF-PVC is more stable than Fe_2_O_3_-PVC [14,15,18]. Furthermore, the first derivative of these prepared composites illustrates the T_max_ having the same considerable trend as T_10_ of prepared samples, which is 321 °C, 311 °C, and 318 °C for pure PVC, Fe_2_O_3_-PVC, and 5% CDF-PVC, respectively, as shown in Figure 13b. Table 4 sums up T_10_ and T_max_ of the synthesized PVC composites.

## 4. Conclusions

The preparation of Cu-doped PVC and Fe_2_O_3_-PVC films leads to good results for the synthesized composites of varying Cu-concentrations. The UV-visible spectrophotometer demonstrates various optical parameters of these synthesized composites, such as transmittance, absorbance, refractive index, optical conductance, electrical conductance, and Urbach energy, revealing the tuned properties of highly Cu-concentrated PVC composites, such as 5% CDF-PVC, while the remaining composites lack excellence compared to 5% CDF-PVD, but they still showed better results than pure PVC. Furthermore, higher thermal stability was noticed for 5% CDF-PVC. In addition, XRD patterns show better interaction of copper-doped iron oxide with the PVC matrix and the presence of vibrational bands corresponding to both components confirms the formation and good aggregation of the CDF-PVC composite. All the mentioned physical and chemical properties of the synthesized PVC-based composite films prove the investigated sample’s efficiency as an attractive material for advanced electronic, optical, and optoelectronic systems, despite their semi-crystalline nature.

## Figures and Tables

**Table 1:** Peak assignment values of FTIR spectra of PVC composites.

Peak Assignments	Pure PVC	Fe_2_O_3_-PVC	1% CDF-PVC	3% CDF-PVC	5% CDF-PVC
Wagging of CH_2_ (cm^−1^)	1438	1438, 2930	1438	1438, 2930	1454
Rocking of CH_2_ (cm^−1^)	961	976	961	976	961
Rocking of C-Cl (cm^−1^)	684	654	-	-	-
Vibration of C-C (cm^−1^)	1084	1069	-	-	-
Vibrational peak of PVC (cm^−1^)	2930	2930	2930	-	2930
Cu-OH bonding (cm^−1^)	-	1438	1438	1438	1454
Presence of PVC in NC (cm^−1^)	1254	654, 1254	1254	1254	1269
Stretching in PVC (C-H IN CH-Cl) (cm^−1^)	1254, 1069	1254, 1069	1254, 1069	1254	1269

**Table 2:** Direct and indirect bandgap energies of PVC composites.

Sr No.	PVC Composites	Direct Bandgap Energy (eV)	Indirect Bandgap Energy (eV)
1	Pure PVC	5.15	5.05
2	Fe_2_O_3_-PVC	5.13	4.99
3	1% CDF-PVC	5.1	5
4	3% CDF-PVC	5.11	4.99
5	5% CDF-PVC	4.85	4.84

**Table 3:** Urbach energies of PVC composites.

Sr No.	Samples	E_0_ (Urbach Energy) eV
1	Pure PVC	2.21
2	Fe_2_O_3_-PVC	2.66
3	1% CDF-PVC	4.07
4	3% CDF-PVC	4.33
5	5% CDF-PVC	4.5

**Table 4:** T_10_ and T_max_ of the synthesized PVC composites.

Sr No.	Sample ID	T_10_ (°C)	T_max_ (°C)
1	Pure PVC	267	321
2	Fe_2_O_3_-PVC	248	311
3	5% CDF-PVC	263	318

## Data Availability

The data used in this study is included in the main text.
